# Audiological Manifestations in Vitiligo Patients

**Published:** 2012

**Authors:** Parvane Mahdi, Masomeh Rouzbahani, Amin Amali, Samad Rezaii Khiabanlu, Mohammad Kamali

**Affiliations:** 1 *Department of audiology, Faculty of rehabilitation sciences, Tehran University of Medical Sciences, Tehran, Iran*; 2 *Otorhinolaryngology Research Center, Imam Khomeini Educational Complex Hospital, Tehran University of Medical Sciences, Tehran, Iran*; 3 *Department of dermatology, Imam Khomeini Educational Complex Hospital, Tehran University of Medical Sciences, Tehran, Iran*; 4 *Department of Rehabilitation Research Center, Faculty of rehabilitation sciences, Tehran University of Medical Sciences, Tehran, Iran*

**Keywords:** Auditory brainstem response, Hearing loss, Pure tone audiometry, Vitiligo

## Abstract

**Introduction:**

The hallmark of vitiligo is the disappearance of melanocytes from the skin due to an as yet unidentified mechanism. The presence of melanocytes in the auditory apparatus suggests that this system could possibly be affected by vitiligo, which targets the melanocytes of the whole body and not just the skin. The purpose of this study was to assess the incidence of auditory alterations in patients with vitiligo

**Materials and Methods:**

A total of 21 patients diagnosed with vitiligo were enrolled in this study. A group of 20 healthy subjects served as a control group. Pure tone audiometry (PTA) and measurements of auditory brain stem responses (ABR) were carried out in all participants.

**Results:**

High frequency sensory neural hearing loss was detected in 8 patients (38.09%). Analysis of ABR revealed that 10 patients (47.61%) had an abnormal increase in the latency of Wave III and 6 (28.57%) had an abnormal prolongation of the inter peak latency between Wave I and III. There was no correlation between age, duration of disease, and any of the recorded parameters.

**Conclusion:**

This study highlights the involvement of the auditory system in patients with vitiligo, suggesting that vitiligo is a systemic disease rather than a purely cutaneous problem.

## Introduction

Vitiligo (leukoderma) is an acquired, sometimes familial, depigmentary disorder which is a result of the selective destruction of melanocytes. It is characterized by pearl-white patches of skin of diverse shapes and sizes in the midst of normally pigmented skin (1-3). Vitiligo affects all races and both sexes equally and has a worldwide occurrence of 0.3 to 1% (4). In Iran, there is no accurate published data; however, it has been estimated that 0.9 to 1.2% of the total population suffers from vitiligo (5). 

From an evolutionary viewpoint, the embryonic origin of human melanocytes is the neural crest and they are located in the epidermis, the hair bulbs of the skin, the uveal tract, the retinal pigmented epithelium of the eyes, the leptomeninges, and the inner ear (4,6). The presence of otic melanocytes was first described by Alphonse Corti in 1831 (3), and these cells are primarily located throughout the stria vascularis and modiolus of the cochlea, but they also exist in the vestibular organs (6-9). Melanocytes may have an important role in the inner ear as hearing is affected in systemic disorders that affect pigmented areas, such as Vogt-Koyanagi and Waardenberg syndromes (3). 

The presence of melanocytes is not limited to the peripheral auditory system, however, as abnormalities in the brainstem have been found in both animals and humans with pigment disorders. Additionally, neurons in the medial superior olivary nucleus of albino rabbits have been shown to be 24% smaller than in normal animals and the branching density of the dendrites to be significantly reduced (10), indicating that melanocytes are also present in the central auditory system. 

Evaluation of auditory function in patients with vitiligo has been the subject of only a few studies and a variety of abnormalities have been reported. In this regard, the present study was designed to use conventional pure tone audiometry (PTA) and measurements of auditory brainstem responses (ABR) to detect any auditory involvement in these patients. 

## Materials and Methods

A cross sectional study was performed in 21 patients with vitiligo (12 female and 9 male) who attended a dermatology clinic in a university hospital center between June 2010 and July 2011. A total of 20 healthy subjects without any history of auditory or vestibular symptoms (10 female and 10 male) participated as control group. The patients all had a definitive diagnosis of vitiligo according to their medical history and a clinical examination verified by a dermatologist. Vitiligo can be classified into 7 clinical types: 1) generalized, 2) focal, 3) segmental, 4) acrofacial, 5) total, 6) inflammatory, and 7) occupational (11). The numbers (percent) of patients who presented with each type were 12 (57.14%), 5 (23.81%), 3 (14.29%), 0 (0%), 1 (4.76%), 0 (0%), and 0 (0%), respectively. A questionnaire containing demographic data, time of disease onset, as well as history of auditory and vestibular problems was completed for each participant.

The following exclusion criteria for both patient and control groups were applied during participant selection: 1) a history of any of middle ear disease, previous ear surgery, familial hearing loss, oral ototoxic drug or corticosteroid intake, chronic noise exposure, head trauma, metabolic, neurological, vascular, or autoimmune disease; 2) the presence of any systemic disease such as diabetes or hypertension; 3) abnormalities in the results of an otoscopic examination; or 4) an age greater than 46 years.

The study protocol was approved by the Ethics Committee of Tehran University of Medical Sciences and all subjects gave written informed consent.

*Pure tone audiometry:*

After an otoscopic examination, participants under went audiometric testing using a Madsen Orbiter 922 diagnostic audiometer (Madsen Electronics, Denmark) at frequencies of 0.25, 0.5, 1.0, 2.0, 4.0, and 8.0 kHz for air conduction and between 0.25 and 4.0 kHz for bone conduction. Pure tone averages (PTA) were calculated at frequencies of 0.5, 1, 2, and 4 kHz; normalcy was based on the criteria of the American Speech-language-Hearing Association (ASHA, 1978) of a threshold below or equal to 20 dBHL (12).

*ABR:*

ABR were measured using an ICS Charter device (GN Otometric, Denmark). Three electrodes were placed one on each of the vertex (noninverted), the ipsilateral mastoid (inverted), and the contralateral mastoid (ground). Interelectrode impedance was kept below 5 kΩ. The acoustic stimuli were rarefaction clicks presented at a repetition rate of 13.1/sec, at an intensity of 80 dB normal hearing level (nHL), with a 0.1 ms duration, delivered monaurally to TDH 39 earphones. Responses to 2048 clicks were preamplified and bandpass filtered between 100 and3000 Hz. The analysis time of the screen was 10 ms. Duplicate recordings were made to check reproducibility. Absolute latency values of Waves I, III, and V and inter peak latencies (IPL) of I–III, III–V, and I–V in each ear were compared with the results of the control group.

*Statistical analysis:*

SPSS software, version 11.5 (Chicago, IL, USA), was used for statistical evaluation. To compare the results from the patient and control groups, a non-parametric test was used (Mann-Whitney *U*-test) as is appropriate for small samples where the data are not normally distributed. The criterion for statistical significance was defined as P≤0.05. Pearson’s correlation was used to find significant relationships between two numeric variables.

## Results

The mean (± standard deviation) age of the patients was 30.50 (±8.05) years (range, 19.75–44.16 years) and for the control subjects it was 31.81 (±8.07) years (range, 18.58–44.91 years). There was no statistically significant difference between these two groups according to sex and age (P > 0.05). The mean duration of the disease in patients was 9.62±8.37 years (range, 0.5–30 years) and 9 patients (42.85%) had a positive family history of disease.

The results of the audiometric tests are shown in (Table 1). 

**Table 1:** Comparison of pure tone audiometry results in patients with vitiligo and control subjects.

**Ear/Frequency(Hz)**	**Patient group (n=21)**	**Control group (n=20)**	**P**
	***Mean ***±**SD(dB) **	*** Mean ***±**SD(dB)**	
R250	8.57±3.91	9.25±3.35	0.471
L250	9.05±4.36	10.00±4.58	0.471
R500	8.33±2.41	7.50±4.13	0.122
L500	8.57±3.58	7.75±3.43	0.438
R1000	8.10±3.34	7.00 ±2.99	0.260
L1000	*7.86*±4.05	8.00±2.99	0.588
R2000	9.76±5.11	6.50±2.35	0.021*
L2000	11.90±9.01	7.00±3.40	0.049*
R4000	17.14±12.50	8.50±4.32	0.005*
L4000	14.76±10.89	8.00±4.10	0.013*
R8000	15.48±11.71	7.75±4.12	0.002*
L8000	18.09±15.36	10.50± 4.83	0.041*
R PTA	10.83±3.79	7.50±2.92	0.003*
L PTA	10.77±5.20	7.68±2.54	0.043*

*Statistically significant

Audiometry thresholds were statistically greater in both ears of the patients with vitiligo at 2, 4, and 8 kHz compared with control subjects (P≤0.05).

Abnormal pure tone thresholds were found in 8 patients (38.09%) including 5 patients with generalized, 1 with total, and 2 with segmental vitiligo, of whom 3 had unilateral and 5 had bilateral sensory neural hearing loss.

(Table 2) compares the ABR findings from patients with vitiligo and control subjects. A statistically significant increase in the absolute latency of the third peak in both the left and right ears (P=0.022 and P=0.001, respectively) was found in the patients. Of the 21 patients with vitiligo, 10 (47.61%) had an abnormal increase in latency of Wave III (greater than 2.5 times the standard deviation of the mean value in normal control subjects), and an abnormal increase in IPL of I–III was also detected in 6 patients (28.57%). However, differences in other peak latencies and IPLs did not reach a statistically significant level. As expected, an increase in the absolute latency of Wave III without any significant changes in the absolute latency of Wave V contributes to a decrease in the IPL of III–V, a finding that was demonstrated in 2 patients (9.25%).

**Table 2:** Comparison of ABR parameters in patients with vitiligo and control subjects

	**Patient group (n=21)**	** Control group (n=20)**	
** Ear/Variable**	***Mean ***±**SD(ms)**	*** Mean ***±**SD(ms)**	**P**
R/ I Latency	1.50±0.12	1.47±0.10	0.432
L/ I Latency	1.50±0.13	1.43±0.14	0.191
R/ III Latency	3.63±0.25	3.45±0.10	0.022*
L/ III Latency	3.62±0.17	3.44±0.11	0.001*
R/ V Latency	5.46±0.26	5.42 ±0.16	0.629
L/ V Latency	5.44±0.21	5.39±0.17	0.410
R/IPL I-III Latency	2.18±0.27	1.99±0.13	0.009*
L/IPL I-III Latency	2.11±0.11	2.01±0.15	0.033*
R/IPL III-V Latency	1.83±0.14	1.96±0.12	0.005*
L/IPL III-V Latency	1.82±0.13	1.94±0.19	0.007*
R/IPL I-V Latency	3.96±0.22	3.81±0.63	0.990
L/IPL I-V Latency	3.93±0.18	3.95±0.19	0.506

## Discussion

Although melanocyte loss in vitiligo is predominantly confined to the patient’s skin, alterations in extracutaneous sites have been reported and sometimes implied for the inner ear along with an associated compromise in function (6). The exact functions of otic melanocytes are not known. They do not appear to be essential for normal hearing but these pigments are assumed to play a protective role against environmental damage (13).

Murillo Cuesta and colleagues (13) showed that, compared with pigmented mice, albino mice show a higher prevalence of age-related hearing loss and poorer recovery of auditory thresholds after getting exposed to noise.

The pattern of hearing loss in our study was as follows: in 3 patients (14.28%) loss was in the range of 2 to 8 kHz, in 3 patients (14.28%) it was limited to 4 to 8 kHz, and in 2 patients (9.52%) it was limited to 4 kHz only. The mean PTA was found to be statistically greater in the right and left ears of patients with vitiligo (P=0.003 and P= 0.043, respectively). Our findings strengthen the hypothesis that an alteration of the inner ear pigment cells might favor the occurrence of hypoacusis. There are discrepancies in the literature about the specific influence of vitiligo on auditory thresholds. Some authors state that vitiligo influences hearing (3, 4, 7, 10, 14–17) whereas others question such influence (8,18). It is likely that differences among the studies in both methodology and subjects characteristics account for the reported variability.

In addition to measurements of hearing loss, some studies have addressed ABR in patients with vitiligo. The sequences of peaks in an ABR recording reflect the synaptic activity of consecutive nuclei along the afferent auditory pathway in the brainstem. Abnormalities in ABR recordings in the present study consisted of an increase in Wave III latency and in the IPL of I–III. Usually Wave III is associated with neural activity that mainly originates from the superior olivary complex (SOC) within the brainstem. The increased IPL of I–III can be explained as being due to abnormal synaptic activity and transmission of the action potential from the auditory nerve to the lower brain stem (4,18). 

In accordance with our results, Elsaied and colleagues (18) also noted a statistically significant increase of the IPL of I–III in patients with vitiligo compared with control subjects. Similarly, other investigators, such as Aydoghan and colleagues (4), found statistically significant increases in Wave III latency and IPL of I–III in both ears and a significant increase in Wave V latency in the right ear of patients with vitiligo compared with control subjects. They suggest that this finding might be a result of delayed synchronization of action potentials in the brainstem nuclei. These results are in contrast to a study by Shalaby and colleagues (8), who found no statistically significant differences between patients and control subjects in any ABR parameters, and they recommended the use of postmortem histopathological studies of the inner ear and brainstem to provide more accurate knowledge of the changes present in patients with vitiligo.

There was no correlation between age, duration of disease, and any of the abnormally recorded parameters (P>0.05), as has previously been shown by Sharma and colleagues (14) and Elsaied and colleagues (18). This may be explained by the possibility that otic melanocytes are affected at the start of the vitiligo and then stabilize afterwards. This suggestion is in contrast with studies by Aslan and colleagues (7) and Ardie and colleagues (10) who found a statistically significance association between the duration of vitiligo and hearing loss.

## Conclusion

In summary, we have presented evidence that implies that as a result of the presence of melanocytes in the auditory apparatus this system shows involvement in vitiligo, and indicates that the disease probably targets the melanocytes of the whole body. Accordingly, patients with vitiligo can be evaluated with pure tone audiometry and ABR measurements even if they do not exhibit any hearing difficulties. For a complete auditory evaluation in these patients further studies should include a larger sample size and the application of other tests such as electrocochleography (EcochG) and otoacoustic emission (OAE) measurements. 
